# On Chloroform

**Published:** 1848-05

**Authors:** Daniel Harrington

**Affiliations:** Dentist, Chestnut street, Philadelphia


### *Chloroform.*

I have administered the ether and the chloroform in a limited number of cases, and in each case, with perfect success, void of any unpleasant effect. In giving the latter to a few persons who were desirous of experiencing its effects upon their own systems, merely for the purpose of experiment, three of the number were affected with the late epidemic influenza, and, apart from any such expectation, each one was found to be perfectly relieved on the influence of the chloroform subsiding, from the convulsive or sneezing propensity, and likewise from the constant, troublesome, watery effusion from the nares; in a word, from every symptom of the disease as having existed *twenty minutes previous to the inhalation* of this singular agent. I have some other cases of cure, of a similar character, so far as relates to the operation of chloroform, that may be communicated at a future period, should they be thought worthy of publication.

Daniel Harrington, Dentist,
Chestnut street, Philadelphia.

